# A Case of Bilateral Cerebral Venous Sinus Thrombosis: More Than Meets the Eye

**DOI:** 10.7759/cureus.26917

**Published:** 2022-07-16

**Authors:** Jordan Valenta, Nazanin Sheikhan, George Trad, Matthew Brockway, Ann Wierman

**Affiliations:** 1 Internal Medicine, Mountainview Hospital, Las Vegas, USA; 2 Hematology/Oncology, Mountainview Hospital, Las Vegas, USA

**Keywords:** hereditary protein s deficiency, cerebral venous sinus thrombosis (cvst), covid-19, plasminogen activator inhibitor-1 (pai-1), jak 2 mutation

## Abstract

Cerebral venous sinus thrombosis (CVST) is a rare etiology of stroke that results from inherited and/or acquired conditions, which can present in a variety of symptoms. CVST in the setting of the 2019 coronavirus disease (COVID-19) has rarely been observed. Herein, we present the case of a 32-year-old female with a recent history of COVID-19 subsequently found to have CVST involving bilateral transverse sinuses. Further workup demonstrated several hypercoagulable conditions, which were likely exacerbated by the viral infection. This case demonstrates an atypical outcome for young, COVID-19-positive patients, which emphasizes the importance of diligence when examining symptomatic patients with a history of COVID-19 infection. The patient was treated with apixaban therapy with radiographic resolution of bilateral CVST and improved vision.

## Introduction

CVST is caused by blood clots in the brain’s veinous systems, which can be provoked or unprovoked, and their clinical presentation can vary depending on location and size [1]. CVST can present as headache, papilledema, visual loss, focal neurologic deficits, seizures, visual loss, altered mental status, and coma. Common causes of CVST include inherited coagulopathy, malignancy, oral contraceptive use, pregnancy, infection, or trauma.

Coronavirus disease 2019 (COVID-19) can present with a wide variety of clinical manifestations, including a hypercoagulable state leading to both arterial and venous thrombosis. Although this viral infection is known to frequently lead to venous and arterial thromboembolic complications, there is growing data and reports suggesting a connection to CVST [2,3]. While this connection continues to be strengthened by research and reports, there is still minimal data on the specific effects of COVID-19 on a patient with previously acquired and/or inherited coagulopathy disorders. 

Among the inherited conditions commonly known to increase the risk of CVST are mutations of the plasminogen activator inhibitor-1 (PAI-1) gene. PAI-1 is a glycoprotein synthesized in endothelial cells, hepatocytes, and adipocytes, which can also be released from activated platelets [4]. PAI-1 is the main regulator of fibrinolytic system activation, inhibiting tissue-type plasminogen activators (tPA) and urokinase-type plasminogen activators (uPA) [4]. Mutations in this gene cause increased activity, leading to inhibition of the natural fibrinolytic system, which in turn leads to hypercoagulability.

There are many documented cases demonstrating the efficacy of anticoagulation in CVST, but there are very few cases that address the efficacy of a typical anticoagulation course in patients with both inherited and acquired hypercoagulable conditions [5]. This lack of documented cases can cause uncertainty and doubt in the minds of providers as to whether typical anticoagulation courses are sufficient or not. In this case presentation, we observe that the use of therapeutic dosing of the direct oral anticoagulant, Apixaban, is sufficient in the treatment of CVST, even in the setting of additional coagulopathies. 

## Case presentation

A 32-year-old African American female with essential hypertension and COVID-19 infection one month prior presented to our emergency department after the loss of consciousness with new-onset seizures. She reported headache, left-eye pain, and blurry vision for the one-week duration. Despite being prescribed medication for her essential hypertension, the patient did not take any home medications or supplements. On initial presentation, vital signs were: body temperature 97.8 F, blood pressure 180/109 mmHg, heart rate 72 beats per minute, respiratory rate 16 breaths per minute, and oxygen saturation 100% on ambient air. Physical examination was notable for right-eye esotropia but intact tracking if left eye covered, as well as sluggish left pupillary response.

Initial laboratory studies, including complete blood count, chemistry panel, and coagulation panel, were notable for a hemoglobin level of 11.2 gm/dL, white blood cell (WBC) of 10.3 k/μL Hematocrit 36.4%, and platelet level of 527 k/μL.

**Figure 1:**
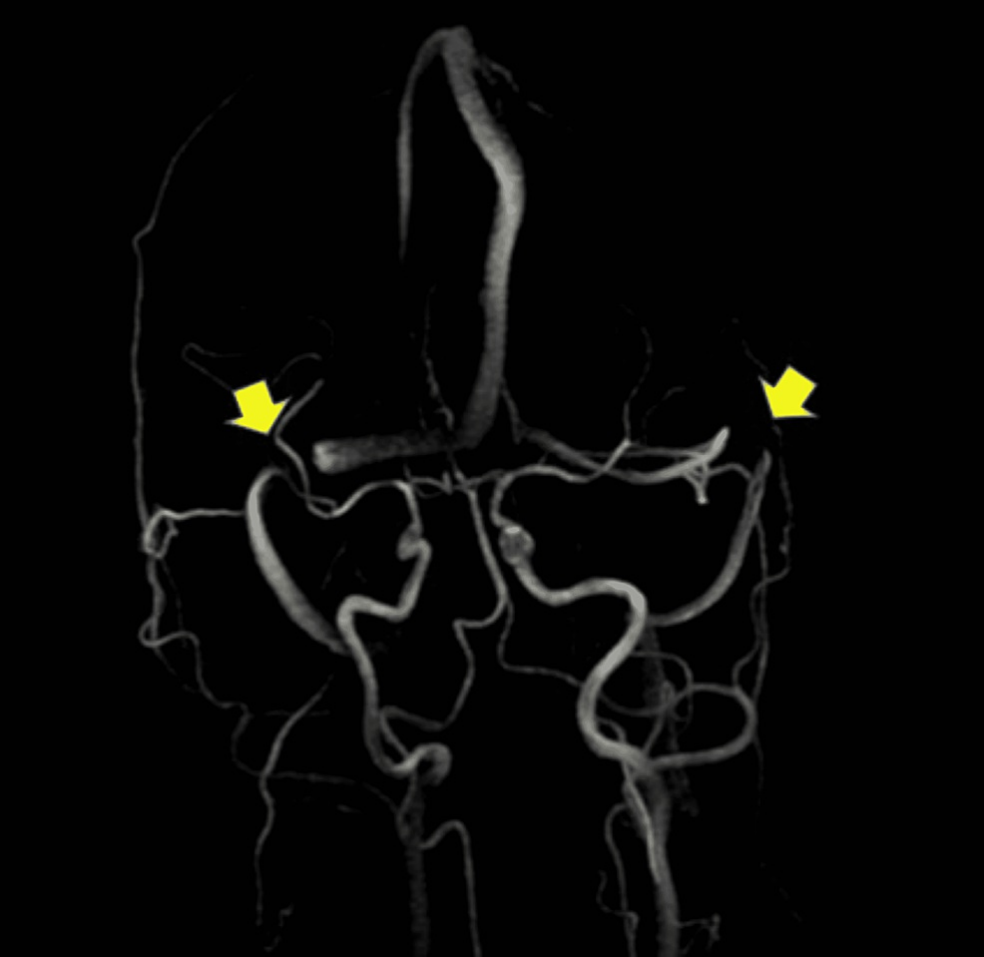
Cerebral MR Venography showing flow gap in the right and left transverse sinus in our patient.

**Figure 2:**
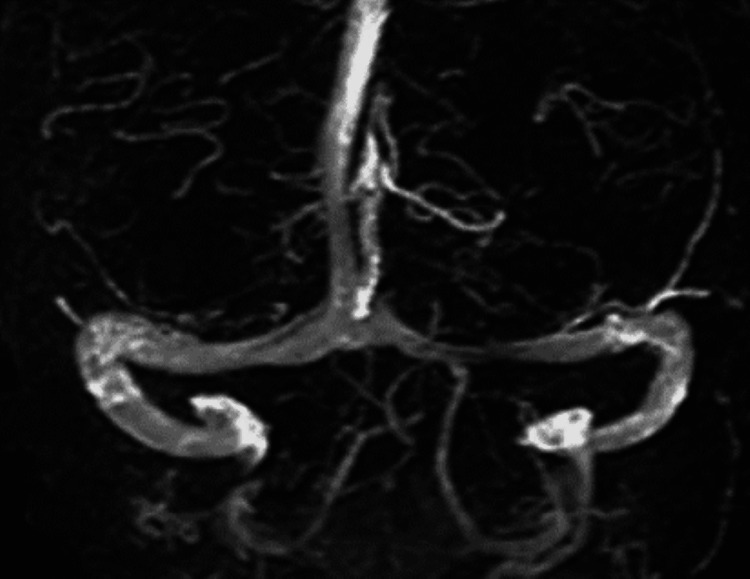
Cerebral MR Venography showing patent blood flow through bilateral transverse sinuses in a patient without CVST R. H. Ayanzen, C. R. Bird, P. J. Keller, F. J. McCully, M. R. Theobald and J. E. Heiserman. Cerebral MR Venography: Normal Anatomy and Potential Diagnostic Pitfalls. American Journal of Neuroradiology January. 2000, 21(1)74-78

Non-contrast computed tomography (CT) of the brain was unremarkable. Magnetic resonance imaging (MRI) of the orbits, face, and neck was normal, ruling out optic neuritis. The MRI of the brain, with and without contrast, demonstrated subtle prominence of the fourth ventricle and cerebellar sulci. The CT angiogram of the head was normal. A magnetic resonance venogram (MRV) of the brain demonstrated venous sinus thrombosis involving the bilateral transverse sinuses. The radiographical findings of the patient's MRV are demonstrated in Figure 1, while the radiographical findings of a clear MRV are demonstrated in Figure 2.

The patient was started on an unfractionated heparin drip and then transitioned to Apixaban on discharge with outpatient hematology-oncology evaluation. Outpatient work-up for thrombophilia yielded Protein S activity deficiency (49%, normal range 60%-130%), Protein C activity (168%), and Protein C antigen (113%), both within normal limits. PCR-based advanced sequencing assay of blood sample showed JAK2V617F mutation, indicative of the presence of myeloproliferative disease. Further genetic analysis revealed Plasminogen Activator Inhibitor-1 (PAI-1) gene 4G/4G polymorphism. After two months of Apixaban therapy, follow-up was notable for improved right eye deviation and visual impairment but persistent imbalance and periodic severe headaches. Subsequent examination and imaging were notable for the resolution of seizures and radiographic resolution of bilateral CVST with repeat MRV. Close follow-up and monitoring are planned with medications, including Apixaban (5mg twice daily), IV iron supplementation for microcytic anemia, and hydrocodone for pain control. The plan of care also included continued ophthalmology examination for right-eye deviation and neurology surveillance for evaluation of seizures.

## Discussion

CVST is a rare yet severe cerebrovascular disease which accounts for 10%-20% of strokes in young adults [6]. The prevalence of CVST is 5 per million, constitutes 0.5% of strokes, and has a mortality rate of just below 10% [7]. The incidence of CVST in COVID-19 patients is 0.02%, which appears low but is 30 to 60 times greater than the incidence of CVST in non-COVID-19 populations. [8]. The annual incidence of CVST in JAK mutation populations has been observed to be 0.26% [9].

Regardless of the etiology of CVST, early diagnosis and management are key to favorable outcomes but can be challenging since the early signs and symptoms of CVST can be non-specific. In this case presentation, we described the history, symptoms, and therapeutic response of our patient to aid clinicians’ awareness of risk factors, symptoms, and warning signs of CVST. This case also adds to the body of literature on CVST as a potential adverse outcome associated with known COVID-19 infection. The patient described in this case had three separate conditions that could contribute to the development of CVST but was only observed to do so one month after contracting COVID-19.

The most commonly reported location for CVST is the superior sagittal sinus, but a recent study suggested that multi-location thrombosis with a transverse sinus predominance is more typical in COVID-19-associated CVST [10]. In the study’s population, it was noted that 62.5% of patients presented with mild to moderate COVID-19 symptoms before or at the time of CVST diagnosis. This finding provides a caution for clinicians as it is evidence that the severity of COVID-19 disease symptoms may not be associated with the likelihood of CVST events.

Traditional CVST management has been a cessation of any present offending agent and anticoagulation for a minimum period of three months or continued indefinitely if a mutation or protein deficiency is involved. There is scarce evidence to support a particular anticoagulant that treats CVST better, but most of the current evidence suggests the use of low-molecular-weight heparin in the acute phase of CVST [11]. However, the open-label RE-SPECT CVT trial demonstrates that patients treated with dabigatran or warfarin had a lower rate of recurrent venous thromboembolism (VTE) events [12]. As COVID-19 has become more widespread, the mainstay treatment for CVST in COVID-19 patients has been therapeutic anticoagulation with adjunct hydration [10]. The use of recombinant tissue plasminogen activators (rtPA) and endovascular therapy is an option but is recommended to be used as a last resort for thrombus refractory to therapeutic anticoagulation or in case of hemodynamic instability and deterioration despite therapeutic anticoagulation [13].

A review of medical literature revealed numerous reported cases of CVST associated with concurrent JAK mutations and PAI-1 gene polymorphisms [1]. However, to our knowledge, our case is the first to investigate the correlation between CVST in the setting of prior COVID-19 infection with three concurrent thrombophilia conditions: JAK mutation, PAI-1 gene polymorphism, and Protein S deficiency. This case illustrates the importance of increased vigilance in patients affected by COVID-19 infection, even if symptoms were mild. Many of the common presenting symptoms of CVST can initially appear non-specific or insignificant, which makes it more essential for clinicians to consider in-depth neurological and vascular imaging in patients that demonstrate irregular and/or persistent symptoms in the setting of past or present COVID-19 infection [4].

Even with the identification of CVST in a COVID-19 patient, our observations suggest that all contributing factors should be explored as viral infection could merely be the initiating factor. An in-depth analysis of patients in a hypercoagulable state should include prompt investigation of underlying acquired and inherited factors, such as JAK mutations, PAI mutations, and Protein S deficiency. This approach to work-up of CVST proved to be particularly significant for this patient as genetic mutations with lifelong implications were identified. By identifying the exact hypercoagulable factors, whether genetic or acquired, the appropriate duration of treatment can be determined. This case report provides crucial information for clinicians, but a shortcoming in our work is that we are unable to provide information on the chance of recurrent VTEs in our patients, which is a common complication of CVST patients.

As evidenced by the continuous improvement of the patient on physical exam and repeat MRI imaging, the standard approach of anticoagulation appears to be sufficient in the treatment of CVST. This case provides more knowledge and confidence to providers to continue using current CVST management recommendations, as our patient improved on this treatment despite multiple contributing factors to a hypercoagulable state.

## Conclusions

Cerebral venous sinus thrombosis is a known adverse outcome of a hypercoagulable state, which can be diagnosed through physical exam findings correlated with radiographic imaging studies (MRV). Although venous sinus thrombosis is uncommonly associated with COVID-19 inflammation, this case report adds to other data to suggest that there should be stronger consideration for such an association. Without treatment, cerebral venous sinus thrombosis can lead to permanent deficits and coma. However, with treatment, this condition has a good prognosis. It is imperative that clinicians understand the association between COVID-19 and cerebral venous sinus thrombosis, especially in the setting of possible genetic factors contributing to hypercoagulability.
